# Nivolumab versus Cabozantinib: Comparing Overall Survival in Metastatic Renal Cell Carcinoma

**DOI:** 10.1371/journal.pone.0155389

**Published:** 2016-06-06

**Authors:** Witold Wiecek, Helene Karcher

**Affiliations:** LASER Analytica, Halton House, 20–23 Holborn, London, EC1N 2JD, United Kingdom; National Institute of Health, UNITED STATES

## Abstract

Renal-cell carcinoma (RCC) affects over 330,000 new patients every year, of whom 1/3 present with metastatic RCC (mRCC) at diagnosis. Most mRCC patients treated with a first-line agent relapse within 1 year and need second-line therapy. The present study aims to compare overall survival (OS) between nivolumab and cabozantinib from two recent pivotal studies comparing, respectively, each one of the two emerging treatments against everolimus in patients who relapse following first-line treatment. Comparison is traditionally carried out using the Bucher method, which assumes proportional hazard. Since OS curves intersected in one of the pivotal studies, models not assuming proportional hazards were also considered to refine the comparison. Four Bayesian parametric survival network meta-analysis models were implemented on overall survival (OS) data digitized from the Kaplan-Meier curves reported in the studies. Three models allowing hazard ratios (HR) to vary over time were assessed against a fixed-HR model. The Bucher method favored cabozantinib, with a fixed HR for OS vs. nivolumab of 1.09 (95% confidence interval: [0.77, 1.54]). However, all models with time-varying HR showed better fits than the fixed-HR model. The log-logistic model fitted the data best, exhibiting a HR for OS initially favoring cabozantinib, the trend inverting to favor nivolumab after month 5 (95% credible interval <1 from 10 months). The initial probability of cabozantinib conferring superior OS was 54%, falling to 41.5% by month 24. Numerical differences in study-adjusted OS estimates between the two treatments remained small. This study evidences that HR for OS of nivolumab vs. cabozantinib varies over time, favoring cabozantinib in the first months of treatment but nivolumab afterwards, a possible indication that patients with poor prognosis benefit more from cabozantinib in terms of survival, nivolumab benefiting patients with better prognosis. More evidence, including real-world observational data, is needed to compare effectiveness between cabozantinib and nivolumab.

## Introduction

Diagnosed in over 330,000 patients, renal cell carcinoma causes over 140,000 deaths worldwide every year [1]. One third of patients have metastatic renal-cell carcinoma (mRCC) at diagnosis [2].

Sunitinib is the most commonly used first-line treatment in mRCC currently, as shown for instance in a retrospective observational study using claims data from the United States where sunitinib was used in 253 out of the total of 273 patients on first-line therapy [3]. However, most patients relapse within 1 year and require a second-line treatment [4], including, among others, everolimus that inhibits the mammalian target of rapamycin (mTOR). Although associated with longer progression-free survival (PFS) than placebo, no significant improvement in overall survival (OS) versus placebo has been demonstrated for everolimus to date [5].

Results from two phase 3 trials on emerging treatments, nivolumab [6] and cabozantinib, [7], used as second-line agent against everolimus for mRCC, were published recently. Nivolumab is a fully humanized lgG4 programmed death 1 (PD-1) inhibitor, whereas cabozantinib is a small-molecule tyrosine kinase inhibitor that targets the vascular endothelial growth factor receptor (VEGFR). Patients from both pivotal trials had received at least one treatment prior to enrolment, with sunitinib, pazopanib and axitinib among the most often prescribed and used by more than 10% of enrolled patients. None of the patients had experienced progression in the 6 months prior to study inclusion, showing a Karnofsky performance status of at least 70% at enrolment. Patients previously treated with an mTOR inhibitor were excluded. The nivolumab trial included 821 patients, as opposed to 658 in the cabozantinib trial. Median overall survival (OS) was 19.6 months (17.6,23.1) in patients on everolimus and 25 months (21.8,NA) for those on nivolumab in the study by Motzer et al [6]; median OS is yet to be reported by Choueri et al due to the low number of deaths at time of data cut-off [7]. The reported hazard ratio (HR) for overall survival endpoint against everolimus was 0.73 (98.5% confidence interval 0.57 to 0.93, P = 0.002) for nivolumab and 0.67 (95% confidence interval 0.51, 0.89, P = 0.005) for cabozantinib.

As no clinical study is currently available that directly compares nivolumab and cabozantinib in mRCC, the objective of the present study was to compare overall survival for cabozantinib and nivolumab in this indication.

## Materials and Methods

The Cochrane Handbook for Systematic Reviews of Interventions recommends synthesizing survival results from different studies using HR [8]. In cases where no direct evidence is available, such being the case here for nivolumab and cabozantinib with only two pivotal studies available in mRCC, indirect comparison of survival data is traditionally performed via a method described in 1997 by Bucher et al [9]. In this method, an HR and its confidence interval for the treatment pair of interest are derived using HRs and confidence intervals from the two original studies against a common comparator. While this method is applicable in the case of nivolumab and cabozantinib OS in mRCC, it supposes proportionality of hazards in each study [10], an assumption that is violated for example when survival curves associated with two treatments intersect. Ouwens et al have proposed a Bayesian network meta-analysis method for indirect comparison of survival endpoints, which does not assume hazard proportionality [11]. As the authors note, using the proportional HR assumption not only has implications on the accuracy of the indirect comparison, it can also have a large impact on any cost-effectiveness analysis that relies on these comparisons. In light of these drawbacks of the standard Bucher method, Bayesian models using different families of distributions were built and assessed to find the model that best fits the data, which subsequently allowed for assessment of comparative effectiveness between cabozantinib and nivolumab.

### Data extraction

**Fig 1:**
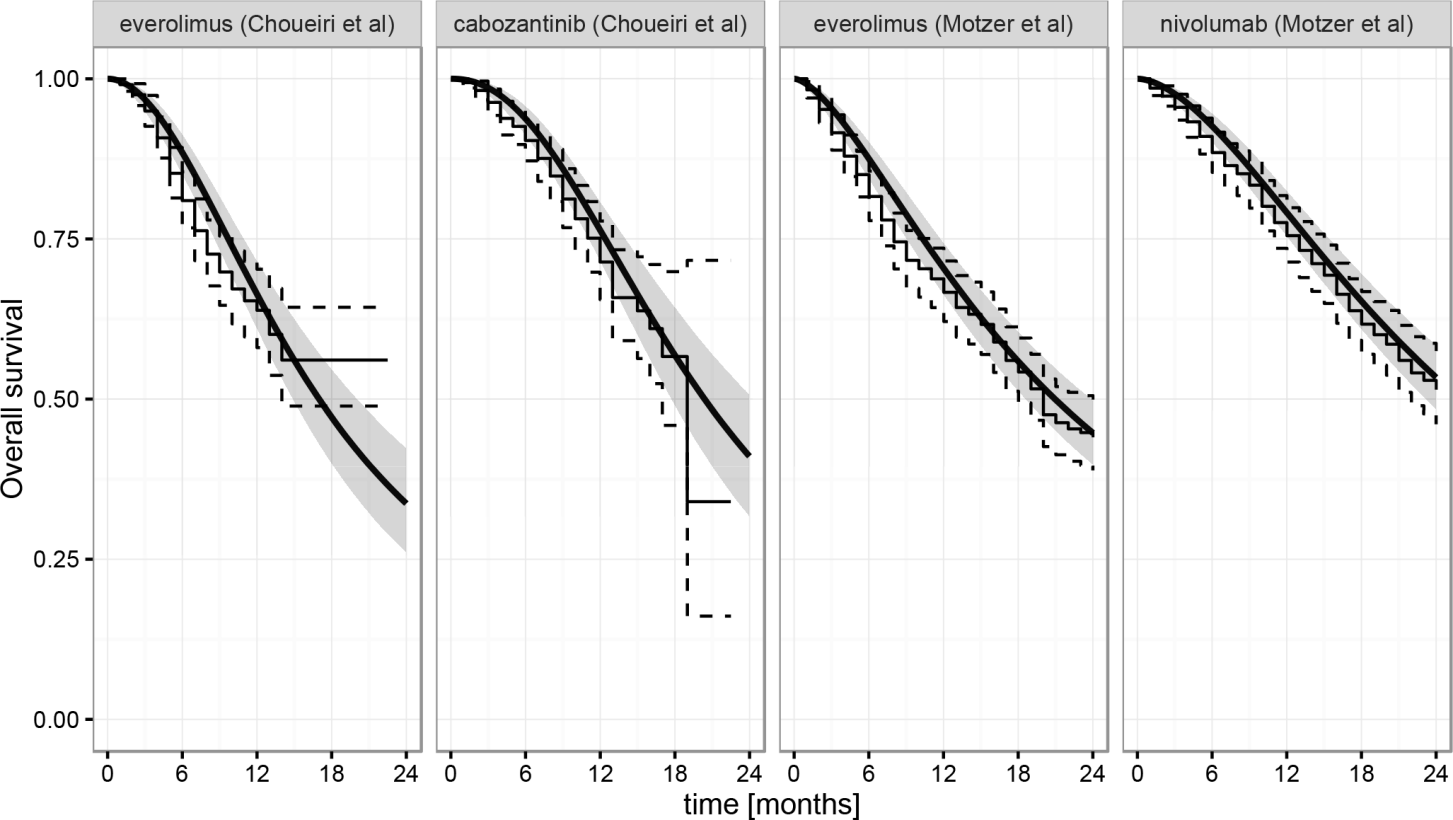
Joint fit of the four overall survival curves that best fit the Bayesian model (log-logistic) overlaid on Kaplan-Meier estimates of data extracted from Choueri et al and Motzer et al [6,7]. Dark smooth line: model estimate, shaded grey area: corresponding 95% credible interval. Thin solid and dashed lines: median and 95% confidence interval for Kaplan-Meier estimates of extracted data.

For each treatment, hazard ratios with confidence intervals, overall survival curves and associated numbers at risk were extracted from two Phase 3 studies published recently (see Figs 1 and 3 from the published work by Motzer et al [6] and Choueiri et al [7]). Applying the method published by Guyot et al [12] to extracted data from the two studies resulted in input tables for the models containing the number of deaths and the number of patients censored every month, for all four survival curves from the studies (see S1 File provided as Supporting Information for the algorithm used for data extraction).

### Statistical analysis

#### Bucher method

First, the HR for nivolumab vs. cabozantinib was calculated as the ratio between reported HRs in the phase 3 studies, that is, the ratio between the HR for nivolumab vs. everolimus against of the one for cabozantinib vs. everolimus. The confidence interval around the calculated HR was derived from the reported confidence intervals around the two HRs resulting from the phase 3 studies (see S1 File for further details on calculations).

#### Bayesian estimation of survival parameters for four families of distributions

Next, an alternative method was used to compare overall survival of nivolumab vs. cabozantinib in mRCC. Namely, a family of parametric distributions was jointly fitted to the data with a Bayesian model, as described by Ouwens et al [11]. This Bayesian approach has the advantage of allowing for estimation of complete distributions of treatment effects for nivolumab and cabozantinib over time, while also enabling calculations of the probability of being the best treatment over time in terms of OS.

More precisely, four models were created each assuming that survival curves for everolimus (two curves), nivolumab (one curve), and cabozantinib (one curve) originated from a specific family of distributions. Three models assumed two-parameter distributions (Weibull, Gompertz, log-logistic) while one model used one-parameter exponential distributions (fixed HR). The latter exponential model was chosen because it made the same assumption as the Bucher method of hazard proportionality. The results were compared against each other and against those calculated using the Bucher formula (see S1 File for more details on how full parametrization was conducted for each of the four models).

Bayesian meta-analysis models were used to determine treatment differences, using everolimus as the reference treatment in each study and estimating cabozantinib and nivolumab in terms of their effect on the reference parameters. Effect transitivity is an underlying model assumption, that is, the models assume that for any time *t*:

$$HR_{nivolumab\;vs\;cabozantinib}(t)=HR_{nivolumab\;vs\;everolimus}(t)/HR_{cabozantinib\;vs\;everolimus}(t)$$

Transitivity implies that the choice of reference treatment in each study has no influence on the results. Note that the Bucher method also assumes this relation, but without allowing the hazard ratios to change with time (see S1 File for a full description of the estimation method).

HR does not depend on which of the two interventions in each study is considered as reference (effect transitivity assumption). However, values of survival functions for nivolumab and cabozantinib over time can only be calculated based on reference values as reported for the everolimus arm of one of the studies. Such adjustment was performed to match the survival values from the everolimus arm in the Motzer et al study [6].

All models were implemented in WinBUGS (see S1 File for a description of the estimation algorithm and convergence criteria used) [13].

Finally, models were ranked for fit assessment according to their (posterior mean) deviance information criterion (DIC), with a lower DIC indicating a better model fit [14]. Both Kaplan-Meier curves generated from extracted data and the four model fits were also visually inspected against original Kaplan-Meier curves from published articles.

## Results

Indirect comparison of hazard ratios using the Bucher method [9] on reported HR and their confidence intervals for nivolumab vs. everolimus and cabozantinib vs. everolimus yielded a HR of nivolumab vs. cabozantinib of 1.09 with a 95% confidence interval of (0.77, 1.54).

Bayesian models allowed for more refined estimates of overall survival over time. The model using log-logistic distributions to jointly fit all four survival curves was the one offering the best fit, with DIC equal to 419.7. Models using Weibull (DIC = 434.4) and Gompertz (DIC = 469.1) distributions both performed better than the one using a constant-HR exponential fit (DIC = 517.2). Potential scale reduction factors did not exceed 1.006 for any of the parameters across the four models, indicating that good convergence was established across the models. Fitted survival curves together with credible intervals for the model exhibiting the best fit are presented as Fig 1 (see S1 and S2 Figs for fitted survival curves for the remaining models). All results for overall survival and hazard ratios of OS are presented for the first 24 months, from which point there were no patients left in the Choueiri study.

**Fig 2:**
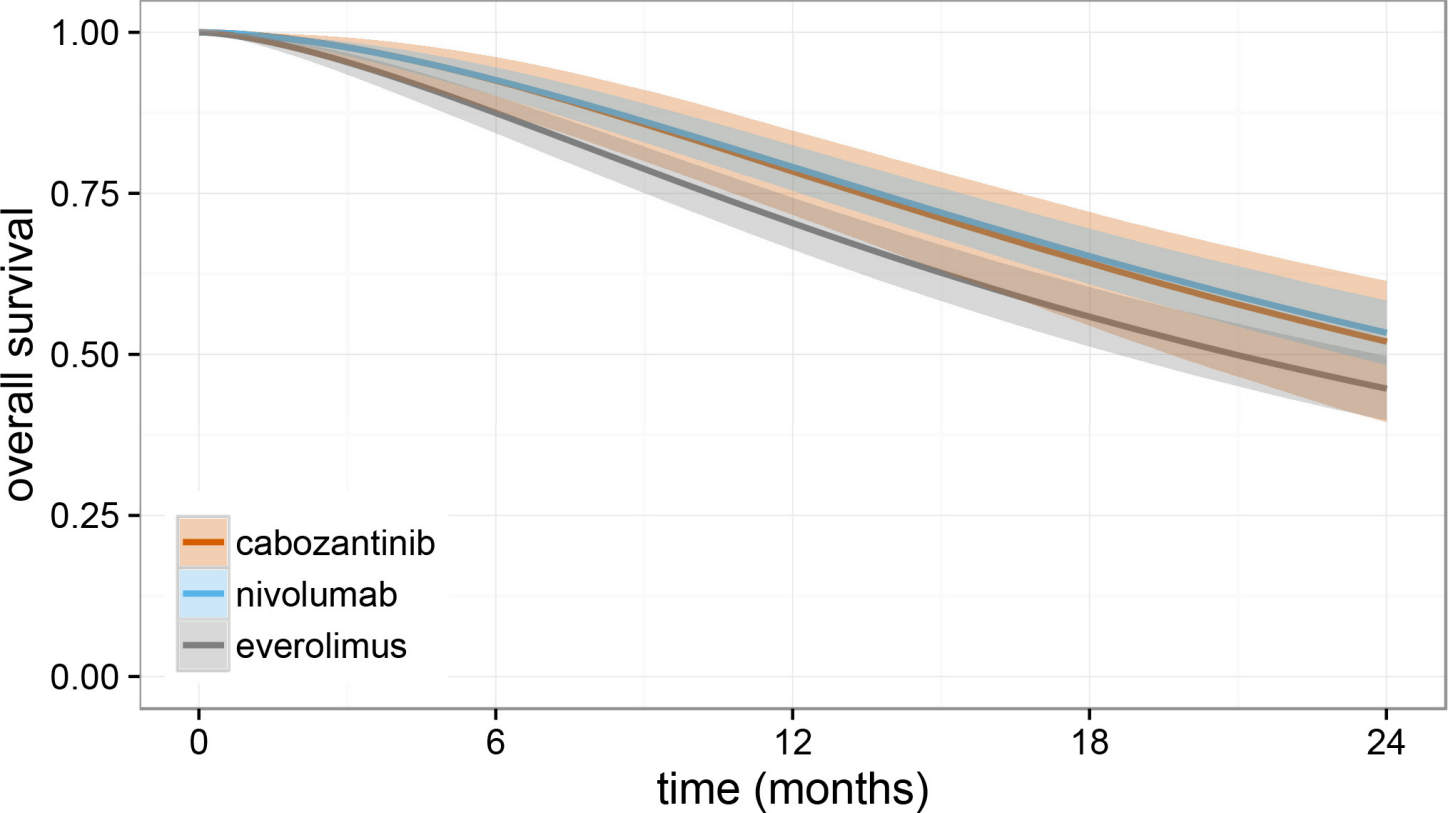
Overall survival curves over time derived from the Bayesian log-logistic model after adjustment of reference parameters to match those in the Motzer et al study [6], Shaded areas represent 95% credible intervals.

Survival curves for nivolumab and cabozantinib were directly compared after adjustment of the reference parameters to match the study by Motzer et al [6], and presented in Fig 2.

**Fig 3:**
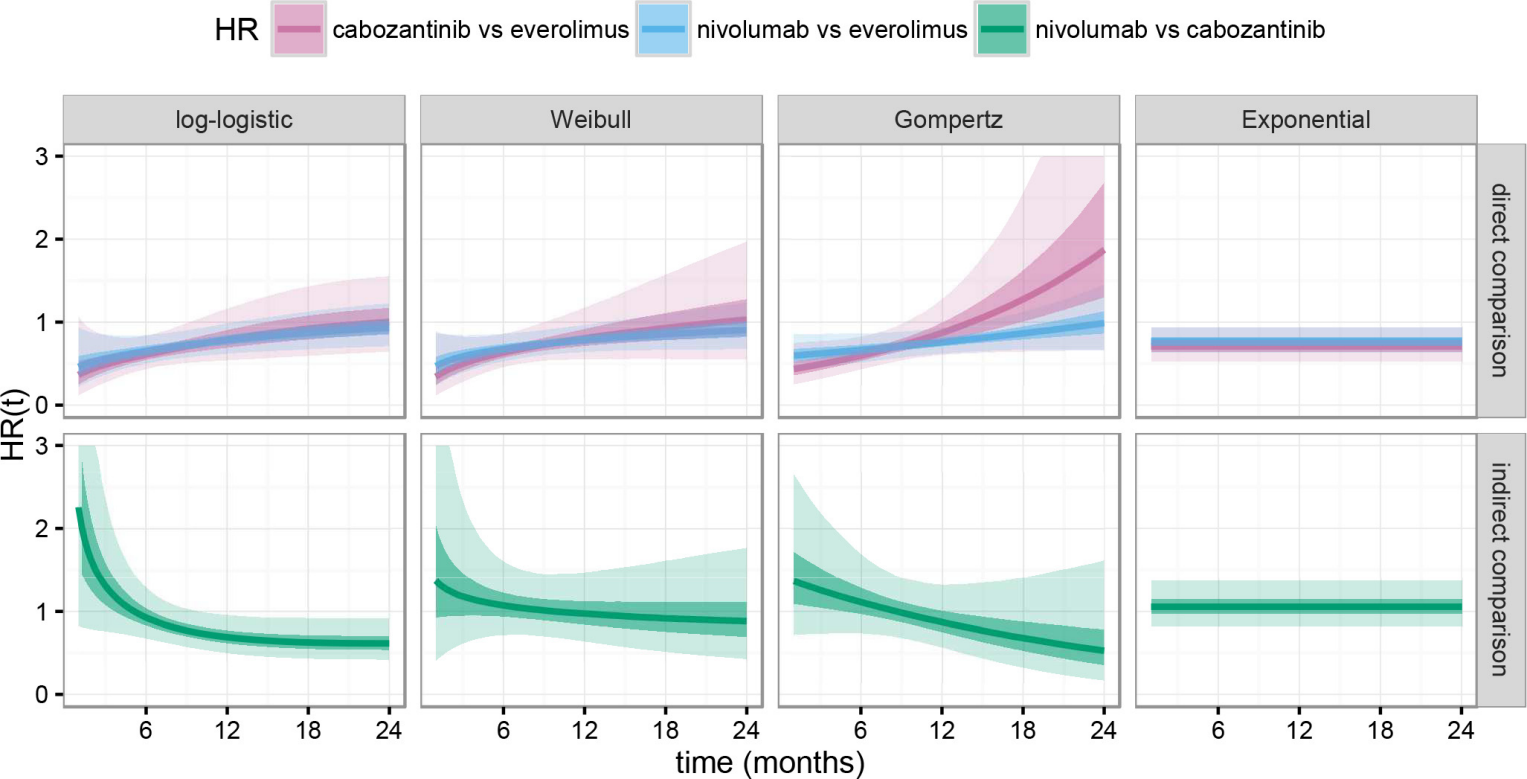
Estimated hazard ratios over time for each treatment pair. Median (solid line), 50% (darker shade) and 95% (lighter shade) credible intervals are represented by model and type of comparison. The log-logistic model (first top and bottom panels to the left) offered the best fit to the data, as opposed to the exponential model (last top and bottom panels to the right) which offered the worst fit.

All three two-parameter models (log-logistic, Weibull, Gompertz) yielded a HR of nivolumab vs. cabozantinib that decreased with time, becoming more favorable to nivolumab as time went by (see Fig 3 and Table 1). The model that best fit the data (log-logistic) resulted in an average HR at month 3 of 1.37 (95% credible interval 0.76, 2.38) dropping to 1 at month 5. From month 10 onwards, the whole 95% credible interval for HR of nivolumab vs. cabozantinib was below 1, which meant that the best model fit yielded a probability of nivolumab having lower hazard than cabozantinib exceeding 97.5% by the second year of treatment. The Weibull and Gompertz models favored cabozantinib at months 3 and 6, with an average HR approaching 1 around month 9 and favoring nivolumab from month 10 onwards. In contrast, the exponential model yielded a time-independent average HR of 1.06, favoring cabozantinib.

**Table 1:** Estimated hazard ratios over time for nivolumab vs. cabozantinib.

	Model-derived HRaverage (95% credible interval)			
Time [months]	Log-logistic DIC = 419.7 (best fit)	Weibull DIC = 434.4	Gompertz DIC = 469.1	Exponential DIC = 517.2 (worst fit)
**3**	1.37 (0.76, 2.38)	1.25 (0.61, 2.34)	1.31 (0.73, 2.19)	1.06 (0.81, 1.37)
**6**	0.94 (0.68, 1.29)	1.09 (0.72, 1.61)	1.14 (0.74, 1.69)	1.06 (0.81, 1.37)
**9**	0.78 (0.57, 1.03)	1.03 (0.70, 1.45)	1.00 (0.68, 1.41)	1.06 (0.81, 1.37)
**12**	0.70 (0.50, 0.96)	0.99 (0.64, 1.47)	0.89 (0.56, 1.32)	1.06 (0.81, 1.37)
**15**	0.66 (0.45, 0.93)	0.97 (0.57, 1.54)	0.80 (0.43, 1.35)	1.06 (0.81, 1.37)
**18**	0.64 (0.43, 0.92)	0.96 (0.51, 1.62)	0.73 (0.32, 1.41)	1.06 (0.81, 1.37)
**21**	0.63 (0.42, 0.92)	0.95 (0.47, 1.70)	0.67 (0.24, 1.51)	1.06 (0.81, 1.37)
**24**	0.63 (0.42, 0.92)	0.84 (0.43, 1.77)	0.62 (0.17, 1.62)	1.06 (0.81, 1.37)

Abbreviations: HR, hazard ratios; DIC, deviance information criterion.

**Fig 4:**
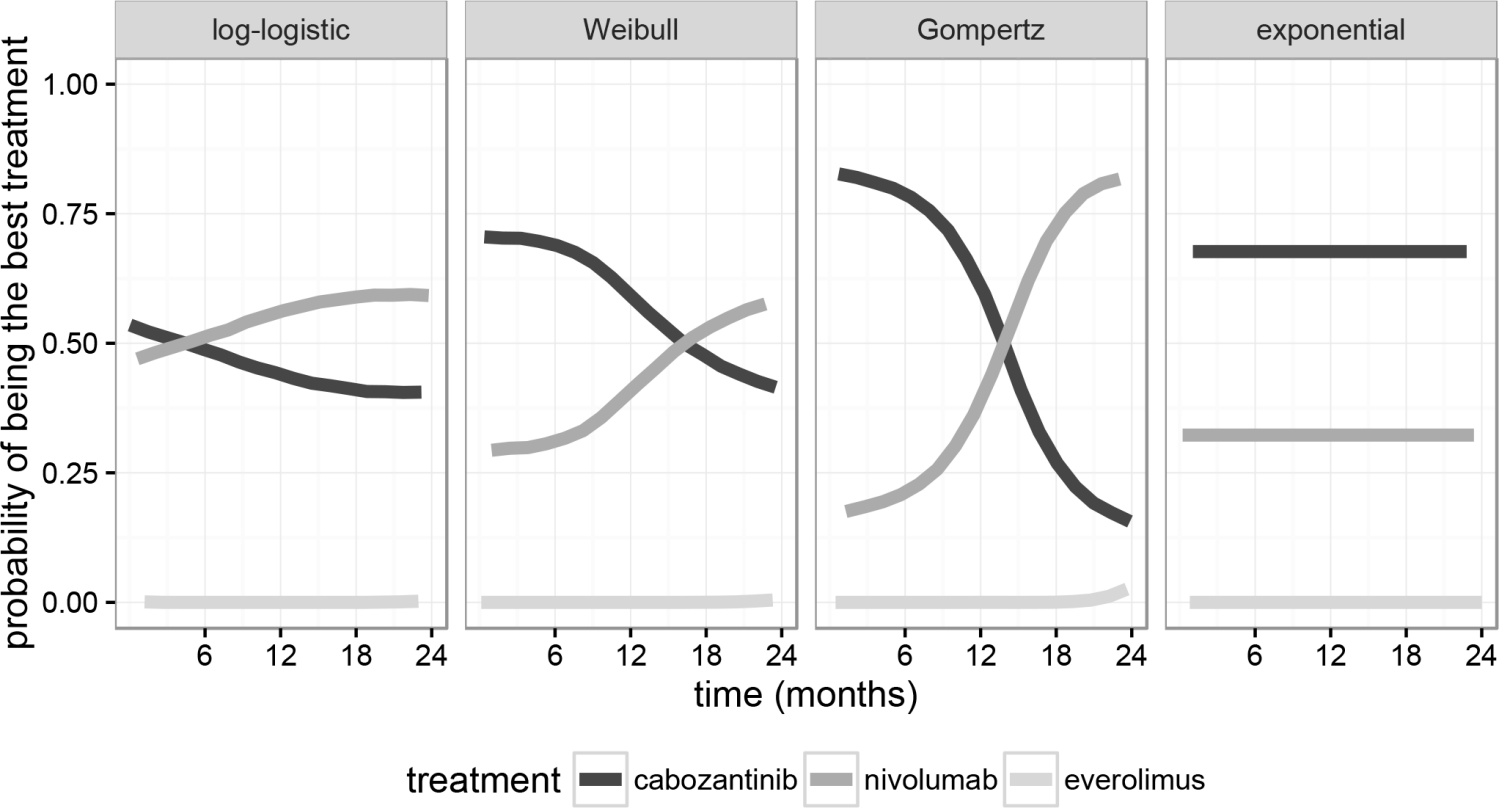
Probability of being the best treatment in terms of overall survival according to the four Bayesian models, as a function of time since the beginning of therapy.

The probability of being the best treatment (in terms of overall survival) was subsequently derived for each treatment and each model as the probability *p* of the survival value at a particular time point for a given treatment exceeding the probabilities established for the remaining interventions (Fig 4). The probability of being the best treatment followed the same pattern across all two-parameter models, with the probability p decreasing for cabozantinib while increasing for nivolumab over time. The log-logistic model (best fit) favored cabozantinib up until month 5, while the Weibull and Gompertz model favored it up until months 16 and 13, respectively. It yielded probabilities of being the best treatment with *p* = 54% in favor of cabozantinib at the start of therapy, changing to *p* = 59.5% in favor of nivolumab at month 24. The odds of being the best treatment [*p*/(1−*p*)] was 1.47 in favor of nivolumab at month 24. The worst fit (exponential model), making the assumption of a constant HR over time, returned a constant probability of being the best treatment of 68% for cabozantinib and 32% for nivolumab (odds of 2.12 in favor of cabozantinib). All models confirmed that the probability of everolimus being the best therapy for overall survival was zero or near zero.

## Discussion

A Bayesian parametric survival network meta-analysis method was applied to compare OS for cabozantinib and nivolumab over time using four families of distributions, in addition to the traditional approach described by Bucher et al [9]. The best model fit showed that patients on cabozantinib exhibited a lower hazard of death of any cause over nivolumab up until the fifth month of treatment, whereas patients on nivolumab exhibited a lower hazard of death of any cause after that period. The assumption of a time-invariant hazard ratio (exponential family model) led to a worse fit than any of the three remaining time-dynamic models considered. All three two-parameter models suggested a similar pattern, showing that the HR of nivolumab vs. cabozantinib decreases over time and also that cabozantinib is preferred at the beginning of therapy (ranging from 5 months for the best log-logistic model, to 10 months for the Weibull and Gompertz models) but favoring nivolumab after that initial period. The analyses conducted in our study highlight the importance of testing comparative methods other than the traditional ones assuming time-invariant hazard ratios. Namely, the (numerically small) benefit of superior OS in later stages of treatment for nivolumab was only evidenced through testing other comparative methods.

A possible interpretation for the lower hazard of death of any cause under cabozantinib in the first five months of treatment for patients who relapsed, the trend inverting to benefit nivolumab for longer treatment duration, is that cabozantinib may be more beneficial than nivolumab in terms of OS when given to patients with a poor prognosis (less than 5 months), whereas nivolumab is more beneficial when given to patients with better prognosis (beyond 5 months). OS prognosis in mRCC is known to depend on many factors (e.g., Heng et al) [15], and several studies have established scores to evaluate prognosis, such as the MSKCC and IMDC [16–19]. In their 2015 study, Motzer et al reported that patients with a poor MSKCC risk score tend to obtain more OS benefit from nivolumab than from everolimus. However, this result was not significant and also not available from the Choueri study for cabozantinib vs. everolimus [7]. It is therefore not possible to verify the interpretation above for nivolumab vs. cabozantinib with the data available to date. This comparative analysis highlights the importance of investigating further if mRCC patients who relapse after first-line treatment benefit more from cabozantinib vs. nivolumab if they have a poor overall survival prognosis, considering switching to nivolumab in those patients with better prognosis. To achieve this, it is vital to report and compare OS in patient groups with different OS prognosis (or risk groups) following treatment with cabozantinib and nivolumab–as expected with the next data release from these trials as well as subsequent trials and cohort studies. Even if direct comparisons are still not available, it will then be possible to evaluate the comparative effectiveness of nivolumab vs. cabozantinib across different populations, and thereby to best position cabozantinib and nivolumab in the mRCC treatment setting.

From a methodological point of view, it is interesting to note that, while this comparison utilizes data from just a couple of studies, the approach can be extended to further comparisons and treatments as the network of studies available grows. The approach could easily be extended by adding other studies comparing the treatments of interest (in this instance, nivolumab or cabozantinib) to either a reference compound (e.g., everolimus) or between the treatments of interest themselves. Also, and more importantly, the approach is equally applicable when adding studies on other treatments, provided the network remains connected, that is, there is always at least one study linking each one of the included treatments to one of the remaining therapies. These features make the method particularly useful to compare indications in rapidly-evolving treatment landscapes, such as mRCC which has seen the approval of novel therapies over the past decade and thereby experienced a profound transformation of its treatment paradigm. In these relatively common cases, the efficacy of new treatments needs to be evaluated against other relatively-new treatments, while it is also likely that (i) direct comparisons are not yet available, (ii) the corresponding network of evidence is star-shaped at best, and/or (iii) study populations are heterogeneous due to the changing nature of the treatment paradigm. The approach used here enables researchers to tackle such situations.

A methodological limitation that may influence the interpretation of the findings is that survival curves originate from the same family of distributions within a same model. Although this is necessary to maintain transitivity property, it is possible that certain OS curves could have been shown to individually best fit other types of distribution besides the log-logistic model. Despite this limitation, the improvement in model fitting obtained with Bayesian models over the standard Bucher comparison was clear.

Another limitation of this analysis results from natural patient censoring in both source studies (less patients with data as the trials progressed), associated with the relatively short minimum follow-up time for OS at the data cut-off date in the Choueri study (6 months against 14 months as reported by Motzer et al) [6,7]. As a result, the total number of patients at risk at 18 months added only to 11 patients in the study by Choueri et al [7]. This means that the uncertainty about true survival curves is increasingly larger as study time progresses, and more so in the Choueri study, as reflected by the growing confidence intervals on fitted survival curves (see Fig 1). It is likely that the analysis presented in this study can be refined in the future, as there are still patients under treatment who have not yet completed the planned treatment period in both studies. Incorporating future data to be released from both studies to the above indirect comparison is expected to add precision to the figures presently reported on OS at later points in time. In the Choueiri et al study, 200 (30.4%) patients continued to receive treatment (including 133 patients treated with cabozantinib), and 95 (11.1%; including 67 patients still being treated with nivolumab) in the study by Motzer et al [6,7]. Despite the data limitations, the modelling method used here demonstrates that the hazard ratio is changing over time in favor of nivolumab vs. cabozantinib. The authors trust this result is likely to hold its validity with the addition of any upcoming remaining trial data from either or both studies as most of the data was included already.

Incorporation of additional data, including real-world observational data, is needed to better define the comparative survival for patients being treated with nivolumab vs. cabozantinib. In particular, it will be interesting to see if and how OS will be affected by the tolerability issues and resulting dose reductions reported so far for patients undergoing longer treatments with cabozantinib as opposed to those being treated with nivolumab, where tolerability do not seem to pose an issue for persistence with the recommended dosage as of yet [20].

## Conclusion

A Bayesian parametric survival network meta-analysis method was applied to compare OS of cabozantinib and nivolumab over time using four families of distributions. All three two-parameter models predicted that HR of nivolumab vs. cabozantinib is favorable to cabozantinib in the first 5 to 10 months and to nivolumab afterwards. Of all four models explored, the one that worse fit the data was found to be the exponential model, which assumes a constant HR over time like in the standard Bucher method, thereby justifying the use of models that do not assume proportional hazards. The model that best fit the overall OS data for both trials was the log-logistic model. In this model, HR for nivolumab vs. cabozantinib returned a numerical estimate <1 (= favoring nivolumab) from month 5 onwards, and full 95% credible interval <1 (= favoring nivolumab) from month 10.

Additional patient data, including real-world observational data, is needed to conclude on comparative survival for patients under treatment with nivolumab vs. cabozantinib. In particular, it is possible that OS for nivolumab will remain superior for longer treatment timeframes in clinical practice as opposed to cabozantinib given the reports on tolerability issues with this latter treatment.

## Supporting Information

S1 Fig Joint fit of four overall survival curves with the best Bayesian model fits from four distributions: log-logistic, Weibull, Gompertz and exponential, overlaid on Kaplan-Meier estimates of data extracted from Motzer et al [S2] and Choueri et al [S3]. Dark smooth line: model estimate, shaded gray area: corresponding 95% credible interval. Thin solid and dashed lines: median and 95% confidence interval for Kaplan-Meier estimates of extracted data.
(EPS)

S2 Fig Overall survival curves over time derived from the 4 Bayesian models after adjustment of reference parameters to match those in the Motzer et al study [S2]. Shaded areas represent 95% credible intervals. The log-logistic model (left panel) offered the best fit to the data, the exponential model (right panel) the worst fit.
(EPS)

S1 File **1) Methods.** a) Algorithm used for data extraction. b) Calculation of the confidence interval around the HR of nivolumab vs. cabozantinib in the Bucher method. c) Parametrization and estimation in each of the four Bayesian models. **2) Results.** S1 and S2 Figs.
(DOC)

S2 File Bayesian models. (ZIP)
