# Supplementary material for: Nivolumab versus Cabozantinib: Comparing Overall Survival in Metastatic Renal Cell Carcinoma
Source: PLoS One. 2016 Jun 6;11(6):e0155389. doi: 10.1371/journal.pone.0155389 (PMC4894561; doi:10.1371/journal.pone.0155389)
Supplement: S1 File — 1) Methods. a) Algorithm used for data extraction. b) Calculation of the confidence interval around the HR of nivolumab vs. cabozantinib in the Bucher method. c) Parametrization and estimation in each of the four Bayesian models. 2) Results. S1 and S2 Figs. (DOC) [file pone.0155389.s003.doc]

# Supporting Information

## Methods

### Algorithm used for data extraction.

The Engauge Digitizer software, version 4.1 (http://digitizer.sourceforge.net/) was used to digitize survival curves with sampling at 1-month intervals on each of the four curves. Subsequently, an existing algorithm[S1] was implemented in the software R, version 3.2.3 (https://cran.r-project.org/) to estimate the monthly number of deaths and the monthly number of censored patients from the digitized curves and the reported numbers at risk. The extraction resulted in input tables for the model containing the number of deaths and the number of patients censored every month, for all four survival curves resulting from the published studies.

### Calculation of the confidence interval around the HR of nivolumab vs. cabozantinib in the Bucher method.

The confidence interval around the HR of nivolumab vs. cabozantinib was derived from the reported confidence intervals around the two HRs in the phase 3 studies using the following mathematical expression:


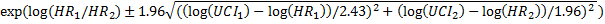


where subscript 1 refers to values from the Motzer et al study[S2] and subscript 2 from the Choueiri et al study;[S3] UCI is the upper confidence interval. The values of 1.96 and 2.43 reflect the fact that a 95% confidence interval was reported in the cabozantinib study, while a 98.5% confidence interval was reported in the nivolumab study (assumption of a normal distribution for reported HR in both studies).

### Parametrization and estimation in each of the four Bayesian models

Each of the four overall survival curves [i.e., everolimus (two curves), nivolumab (one curve), and cabozantinib (one curve)] was parametrized using an underlying hazard function over time, *h(t)*, specific to the model considered:


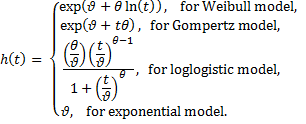


By convention, the
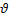
 and
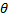
 parameters are called scale and shape, respectively.

Using *j* to index studies (j=1 for Motzer et al,[S2] j=2 for Choueiri et al[S3]) and *k* to index interventions, the two-parameter models can be expressed as


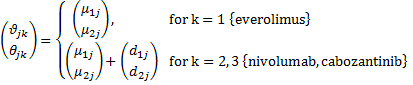


where
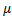
 parameters are the effect sizes for everolimus (scale and shape or just scale in case of exponential distribution) and
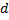
 parameters are the true effect sizes of nivolumab and cabozantinib. It was assumed that
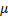
 and
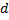
 originated from independent normal distributions (bivariate for two-parameter models, univariate for exponential distribution) – mean and variance of such distributions constitute Bayesian parameters of the model and thus required a choice of prior distributions; this described a fixed-effects model. Please note that it was not possible to implement a random-effects model structure with just two studies.

*Estimation algorithm and convergence criteria*

For each model four chains of length 100,000 (with warm-up of 50,000) were used with uninformative priors for mean and precision of normal distributions (identical choices to the ones used in Ouwens et al).[S4, S5] Convergence was assessed using potential scale reduction factor [S5] with 1.05 chosen as an acceptable threshold value.

## Results. Fig S1 and Fig S2.

**References**

S1. Guyot P, Ades AE, Ouwens MJ, Welton NJ. Enhanced secondary analysis of survival data: reconstructing the data from published Kaplan-Meier survival curves. BMC Med Res Methodol. 2012; 12: 9. doi: 10.1186/1471-2288-12-9.

S2. Motzer RJ, Escudier B, McDermott DF, George S, Hammers HJ, Srinivas S, et al; for the CheckMate 025 Investigators. Nivolumab versus Everolimus in Advanced Renal-Cell Carcinoma. N Engl J Med. 2015; 373: 1803-1813. doi:10.1056/NEJMoa1510665.

S3. Choueiri TK, Escudier B, Powles T, Mainwaring PN, Rini BI, Donskov F, et al; METEOR Investigators. Cabozantinib versus Everolimus in Advanced Renal-Cell Carcinoma. N Engl J Med. 2015; 373: 1814-1823. doi: 10.1056/NEJMoa1510016.

S4. Ouwens MJ, Philips Z, Jansen JP. Network meta-analysis of parametric survival curves. Res Synth Methods. 2010; 1(3-4): 258-271. doi: 10.1002/jrsm.25.

S5. Gelman A, Rubin DB. Inference from iterative simulation using multiple sequences. Stat Sci. 1992; 7: 457-472.
