# Supplementary figures and images for: Nivolumab versus Cabozantinib: Comparing Overall Survival in Metastatic Renal Cell Carcinoma
Source: PLoS One. 2016 Jun 6;11(6):e0155389. doi: 10.1371/journal.pone.0155389 (PMC4894561; doi:10.1371/journal.pone.0155389)

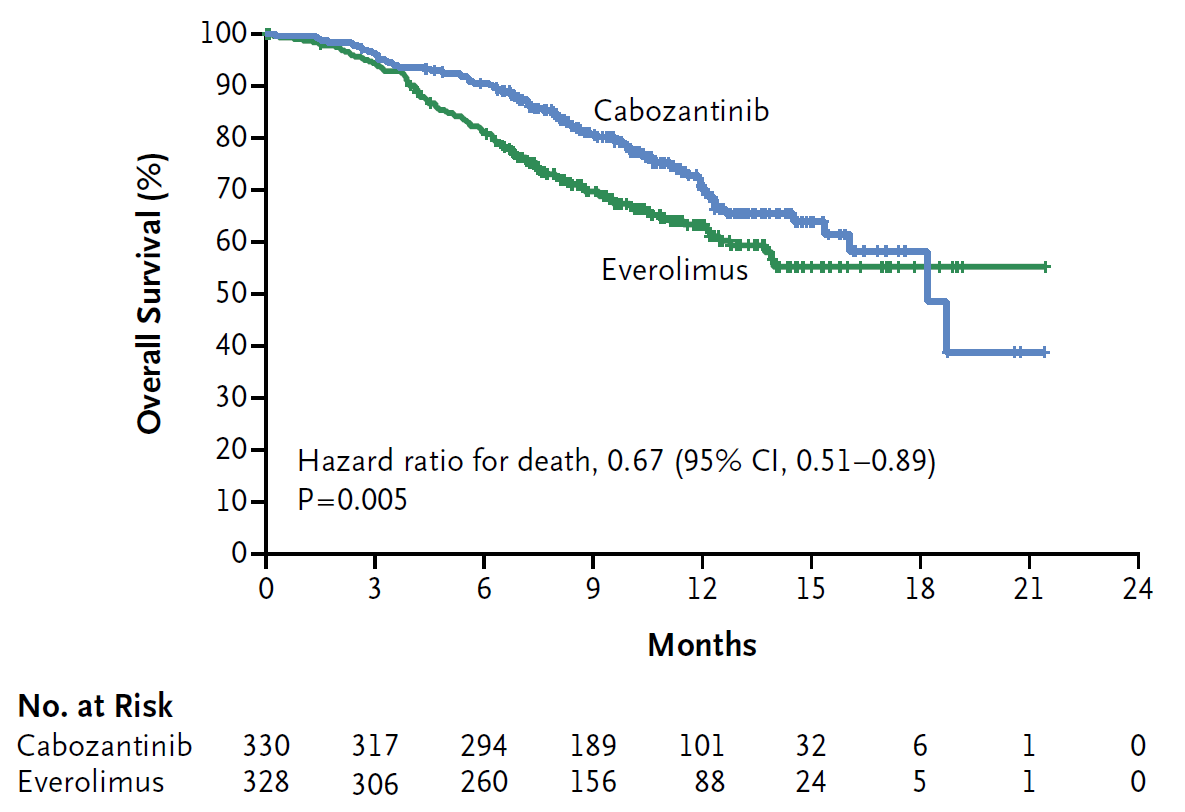

Supplement: S2 File — (ZIP) [file pone.0155389.s004.zip › Bayesian_models/Source_data_2_pivotal_trials/inputs/os1.png]

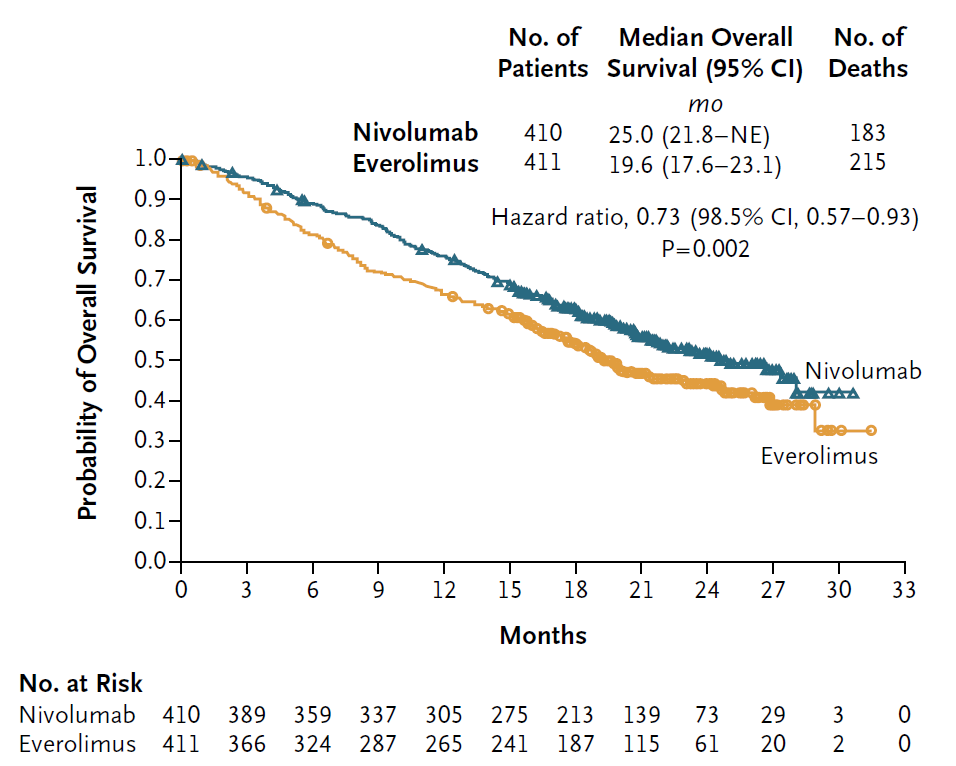

Supplement: S2 File — (ZIP) [file pone.0155389.s004.zip › Bayesian_models/Source_data_2_pivotal_trials/inputs/os2.png]
